# 

**Published:** 1888-09

**Authors:** 


### *To Readers and Correspondents!*

Communications intended for publication, and exchange journals and papers should be addressed to the EDITOR OF THE AMERICAN JOURNAL OF DENTAL SCIENCE, 845 N. Eutaw St., Baltimore, Md. All letters relating to the business of the JOURNAL, or containing cash remittances, to be directed to Messrs. SNOWDEN & COWMAN. Articles for publication should be received by the editor at least three weeks previous to the first month on which the number in which it is designed for them to appear, is issued.

☞ The AMERICAN JOURNAL OF DENTAL SCIENCE will contain fifty pages of reading matter in each number, making a volume of about

*Six Hundred* pages,

EXCLUSIVE OF ADVERTISEMENTS.

